# Divalent metal ions enhance bone regeneration through modulation of nervous systems and metabolic pathways

**DOI:** 10.1016/j.bioactmat.2025.01.034

**Published:** 2025-02-12

**Authors:** Ying Luo, Baoyi Liu, Yashi Qiu, Lichen Li, Fan Yang, Chao Zhang, Jiali Wang

**Affiliations:** a School of Biomedical Engineering, Shenzhen Campus of Sun Yat-sen University, Shenzhen, Guangdong, 518107, China; b Department of Orthopedics, Affiliated Zhongshan Hospital of Dalian University, No. 6 Jiefang Street, Dalian, Liaoning, China

## Abstract

The divalent metal cations promote new bone formation through modulation of sensory and sympathetic nervous systems (SNS) activities. In addition, acetylcholine (Ach), as a chief neurotransmitter released by the parasympathetic nervous system (PNS), also affects bone remodeling, so it is of worth to investigate if the divalent cations influence PNS activity. Of note, these cations are key co-enzymes modulating glucose metabolism. Aerobic glycolysis rather than oxidative phosphorylation favors osteogenesis of mesenchymal stem cells (MSCs), so it is of interest to study the effects of these cations on glucose metabolic pathway. Prior to biological function assessment, the tolerance limits of the divalent metal cations (Mg^2+^, Zn^2+^, and Ca^2+^) and their combinations were profiled. In terms of direct effects, these divalent cations potentially enhanced migration and adhesion capability of MSCs through upregulating *Tgf-β1* and *Integrin-β1* levels. Interestingly, the divalent cations alone did not influence osteogenesis and aerobic glycolysis of undifferentiated MSCs. However, once the osteogenic differentiation of MSCs was initiated by neurotransmitters or osteogenic differentiation medium, the osteogenesis of MSCs could be significantly promoted by the divalent cations, which was accompanied by the improved aerobic glycolysis. In terms of indirect effects, the divalent cations significantly upregulated levels of sensory nerve derived CGRP, PNS produced choline acetyltransferase and type H vessels, while significantly tuned down sympathetic activity in the defect zone in rats, thereby contributing to significantly increased bone formation relative to the control group. Together, the divalent cations favor bone regeneration via modulation of sensory-autonomic nervous systems and promotion of aerobic glycolysis-driven osteogenesis of MSCs after osteogenic initiation by neurotransmitters.

## Highlights

• In terms of the indirect pathway, the divalent cations upregulated activities of sensory nerves and parasympathetic nervous system (PNS) while downregulated activity of sympathetic nervous system (SNS), thereby contributing to increased bone and type H regeneration in the bone defects.
• The divalent cations alone could not initiate osteogenic differentiation of MSCs and affect their aerobic glycolysis, while the osteogenic induction medium (OIM) or neurotransmitters could initiate osteogenic differentiation of MSCs.
• In terms of the direct pathway, after initiation of osteogenic differentiation, the MSCs with the treatment of the divalent cations exhibited significantly higher osteogenesis and improved aerobic glycolysis.

## Graphical abstract

**Figure:**
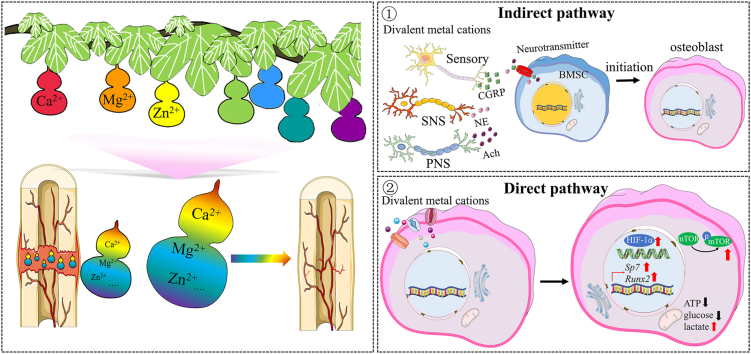

## 1 Introduction

In the last few decades, magnesium (Mg) ions [1], zinc (Zn) ions [2], calcium (Ca) ions [3], and copper (Cu) ions [4], which are mainly stored in muscle and bones as the key-enzymes in over 600 enzymatic reactions [[5], [6], [7]], have been reported to play vital roles in bone regeneration. These divalent cations exert their favorable effects from both direct and indirect pathways, including angiogenesis [8], nerve fiber outgrowth [9], osteoimmunomodulation [10], cell migration and adhesion [11]. The research progress in their biological functions inspire and encourage material scientists and clinicians to develop these metal cations containing hydrogel or scaffold or fixators in orthopaedics for accelerated bone formation [4,12,13]. Most recently, the understanding of the brain-bone axis activated by the divalent metal cations was explored to unveil the underlying mechanism behind the beneficial effects of these cations on bone modeling and remodeling through the unique indirect pathway [1,14]. Different from modulation of angiogenesis-osteogenesis crosstalk [15] located in the *in situ* micro-environment, the regulation of skeletal interoception involves communications among macrophages, peripheral sensory nerves, central nervous systems, and autonomic nervous systems (ANS) [14].

Generally, the ANS is a network of nerves that handle unconscious tasks like the heart rate, blood pressure, breathing, and digestion. The sympathetic nervous system (SNS), as one of the two divisions of the ANS, enables the body to deal with stressors through the "fight or flight" response and maintain homeostasis during daily activities such as exercising, eating, or regulating body temperature [16]. In addition, the SNS regulates immune activity through innervation of immune organs such as the spleen, thymus, and lymph nodes [17]. The parasympathetic nervous system (PNS), as the other one of two divisions of the ANS, promotes the “rest and recovery” process [18], constricting the pupils, slowing heart rate and contraction force, constricting bronchial muscles and stimulating bronchial secretions, and enhancing intestinal motility for efficient digestion [19]. In addition to the internal organs, the ANS has been also reported to play a significant role in bone development, remodeling, and homeostasis in recent years [20]. For instance, increased SNS activity can cause bone loss by increasing bone resorption and decreasing bone formation due to the release of norepinephrine (NE) [21] while the activation PNS promotes proliferation and differentiation of osteoblasts via secretion of the neurotransmitter acetylcholine (Ach) [22].

Of note, the divalent metal cations favor the production of prostaglandin E2 (PGE2), which is released by macrophages and activates EP4 receptors on sensory nerves, thereby leading to upregulation of calcitonin gene-related peptide (CGRP) and downregulation of NE from sensory and SNS, respectively [14]. In addition, the secretion of Ach, a neurotransmitter produced from the PNS regulating bone remodeling [23], is also promoted via activation of PGE2/EP4 signaling axis [24], so it is of worth to investigate if the divalent cations activate PNS for enhanced bone formation.

In addition to the indirect pathway affecting osteogenesis of mesenchymal stem cells (MSCs), accumulating evidence has shown that glucose metabolism is closely associated with osteogenic differentiation ability of MSCs [25]. Interestingly, although cell reliance on oxidative phosphorylation increases over glycolysis after initiation of osteogenic differentiation [26], aerobic glycolysis rather than oxidative phosphorylation favors osteogenic differentiation of MSCs [27]. For example, the upregulation of hypoxic inducible factor 1-α (HIF-1α) or activation of the mammalian target of rapamycin (mTOR) effectively promoted aerobic glycolysis [28,29], ultimately contributing to improved osteogenesis of MSCs [30]. As the divalent cations, including Mg, Zn, and Ca, are critical coenzymes regulating hundreds of major metabolic and biochemical process and also directly upregulate osteogenic differentiation of MSCs in the osteogenic induction medium (OIM) [12,13], the effects of the divalent cations on the metabolism switch from oxidative phosphorylation to aerobic glycolysis needs further investigation.

Herein, we raised a hypothesis that the divalent cations may significantly promote new bone formation through modulation of sensory-sympathetic-parasympathetic nervous systems (indirect pathway) and alteration of the glucose metabolism mode (direct pathway) of the differentiated MSCs. In order to verify this hypothesis, in this study, we investigated the role of the divalent cations and neurotransmitters in osteogenesis and aerobic glycolysis of MSCs with or without initiation of osteogenic differentiation. In addition, we conducted a bone defect model in rats to test if the divalent cations could affect bone regeneration through influencing neurotransmitter production from sensory nerves, SNS, or PNS. Together, our work may lay down a foundation for the elucidation of the repair mechanism of the divalent cations in bone formation through both of the direct and the indirect pathways.

## 2 Materials and methods

### 2.1 Isolation and culture of mesenchymal stem cells from bone marrow in rats

Sprague–Dawley (SD) rats were purchased from the Experimental Animal Centre of Sun Yat-sen University under the approval of the Animal Care and Experiment Committee of the Sun Yat-sen University (SYSU-IACUC-2022-001991). We isolated and cultured the bone marrow derived mesenchymal stem cells according to a well-established protocol [[31], [32], [33]]. First, the rats at age 3 weeks were sacrificed and then shortly immersed in 75 % ethanol prior to collection of femora and tibiae at the biosafety cabinet. Afterwards, the bone marrow was flushed out with the cold PBS by syringe at both the proximal and distal ends of these bone shafts. Then, the collected bone marrow cell suspensions were filtered through a 70-μm cell strainer into a 50 ml tube in order to remove possible bone fragments prior to centrifugation at 1000*g* for 5 min. After that, the cell pellets at the tube bottom were flushed by the complete cell culture medium consisting of Dulbecco's modified Eagle medium/nutrient mixture F-12 (Ham) (Gibco, USA), 10 % fetal bovine serum (Gibco, USA) and 1 % streptomycin and penicillin (Gibco, USA) and plated in the culture dish for culture in a 5 % CO_2_ incubator at 37 °C. The BMSCs at passages 2–4 were used for subsequent experiments. The stock solutions of 1 M magnesium chloride (MgCl_2_), 10 mM zinc chloride (ZnCl_2_), and 1 M calcium chloride (CaCl_2_) (all from Sigma-Aldrich, Germany) were prepared in double-distilled water (ddH_2_O) for adjustment of Mg^2+^, Zn^2+^ and Ca^2+^ concentrations in the cell culture medium.

### 2.2 Cytotoxicity assay

The cytocompatibility of the cell culture medium added with a single ion (Mg^2+^, Zn^2+^ or Ca^2+^) or their combined dual ions at a series of concentrations was assessed using both Cell Counting Kit-8 (CCK-8) and Live/Dead assays. As summarized in Table S1 [[34], [35], [36], [37]], CCK-8, as a high sensitive, stable, and low toxic assay, can measure the number of live cells by determining the activity of dehydrogenases in the cells through quantitative measurement of the absorbance of a dye produced by live cells. Live/Dead cell imaging assay, as a highly sensitive, low toxic, and accurate method, can stain live cells bright green and dead cells red, respectively, based on intracellular esterase activity and plasma membrane integrity. MTT assay, as a rapid method, can quantitatively assess the ability of metabolically active cells through measurement of the amount of formazan. Lactate dehydrogenase (LDH) assay, as a sensitive and low toxic method, is widely used to measure cell viability and apoptosis through detection of the amount of LDH released from damaged cell membranes. However, the MTT assay is more toxic and less sensitive than other methods. In addition, a variety of chemical compounds can interfere with the MTT assay. In terms of LDH assay, its major limitation is that serum and some other compounds have inherent LDH activity. Therefore, we applied CCK-8 and Live/Dead assays for cytotoxicity assessment. 100 μL of the normal complete medium containing 2 × 10^3^ of BMSCs was seeded in each well in a 96-well plate. After 24 h, the stock solutions of metal ions were added into wells to prepare a series of concentrations of a single or combined dual ions. After 72 h of incubation, a CCK-8 kit (CCK8, Biosharp, China) or a Live/Dead double-staining kit (KeyGen, China) was added to detect cell viability through measurement of the absorbance at 450 nm and detection of fluorescence images using fluorescence microscopy (Leica, Germany), respectively.

### 2.3 RNA-sequencing (RNA-seq) and bioinformatics analysis

The total RNA of the cells was extracted and purified using the RNeasy Plus kit (Roche, China) following the manufacturer's instructions. The concentration of RNA was measured with a NanoDrop 2000 spectrophotometer. Two hundred nanograms of total RNA was used for sequencing on the MGI DNBSEQ instrument (MGI, Shenzhen, China). mRNA molecules were then purified using magnetic beads coated with poly-T oligo, which was followed by cDNA synthesis. A single “A” base was subsequently added to the 3-prime end of the synthesized blunt-ended cDNA and ligated with index adapters for hybridization onto a flow cell. The DNA fragments with adapters on both ends were amplified via polymerase chain reaction to generate the final double-stranded cDNA (ds-cDNA) library followed by library validation and normalization and pooling of the samples. The ds-cDNAs were then subjected to heat denatured and circularized by the splint oligo sequence to generate the single strand circle DNA, which was followed by rolling circle replication to create DNA nanoballs (DNB) for processing on the MGI DNBSEQ. The clean reads were filtered from raw reads by FastQC and used for further bioinformatics analysis. The differentially expressed mRNAs were selected based on log_2_ (fold changes) > 1 and *p* value < 0.05. According to the website provided by BGI (https://biosys.bgi.com/), volcano plots demonstrated differential gene expression (DGE) between the two groups. Gene ontology (GO) analysis was performed to explore the molecular function, cellular components and biological processes of the differentially expressed mRNAs. The biological pathways of the differentially expressed mRNAs were further analyzed using the Kyoto Encyclopedia of Genes and Genomes (KEGG) database.

### 2.4 Osteogenic differentiation of BMSCs

Osteogenic induction medium (OIM) is composed of a basic growth medium containing 100 nM dexamethasone (Sigma, USA), 10 mM sodium β-glycerophosphate (Sigma, USA) and 50 μM ascorbic acid (Sigma, USA). BMSCs at passage 2 or 3 were seeded in 12-well or 6-well plates until the cells grew to about 70–80 % confluence. Then, the OIM with or without the addition of the divalent cations at their maximum tolerated concentrations was added to induce osteogenic differentiation of BMSCs. The OIM was refreshed every 3 days. In addition, in order to investigate how the divalent cations exert their favorable effects on osteogenic differentiation of BMSCs, the pre-osteogenic, i.e. without OIM substances, and the post-osteogenic, i.e. with OIM substances as the differentiation initiator, induction models were used. As our previous work confirmed that the Mg^2+^ ions could stimulate the release of CGRP from the sensory nerve fibers [1], thereby promoting osteogenic differentiation of MSCs, CGRP at 10 nM in the pre-osteogenic induction model was chosen as the potential differentiation initiator in this study. In addition, the extracted proteins from the lysed cells were used for Western blot analysis to quantify RUNX2 and SP7 levels.

### 2.5 *In vitro* metabolic assays

The osteoblasts differentiated from BMSCs were seeded and then treated by the medium containing the divalent metal cations for 48 h for glucose consumption, lactate production, and intracellular ATP level measurement. Briefly, glucose consumption and lactate production in the medium were measured with the Glucose Assay kit (Beyotime, S0201M) and the L-lactate Assay Kit (Abbkine, KTB1100), respectively. ATP was measured in cell lysates with a commercial kit (Beyotime, S0026). In addition, the extracted proteins from the lysed cells were used for Western blot analysis to quantify HIF-1α and p-mTOR/m-TOR ratio. In terms of mitochondrial respiration measurement, BMSCs at passage 2 or 3 were seeded in a 6-well plate until the cells grew up to about 70–80 % confluence. The divalent cations were added into the cell culture medium to treat BMSCs at 3 days and 7 days after their osteogenic differentiation in the OIM. After 48 h, mitochondrial respiration of the differentiated BMSCs at 3 days and 7 days was determined by using the mitochondrial respiratory chain complex I activity assay kit (KTB1850, China). In brief, the cells were lysed and then homogenized in a mortar in an ice bath, which was followed by centrifugation at 600*g* and 4 °C for 5 min for the collection of the supernatant. Afterwards, the supernatant was centrifuged again at 11,000 g and 4 °C for 10 min. The precipitate, as the extracted mitochondria, were resuspended in working solution for absorbance measurement at 0 min and 2 min at 340 nm. In terms of glucose uptake, the medium of BMSCs after above treatment was replaced with the glucose-free medium to treat cells for 1 h. After the removal of the glucose-free medium, the 2-NBDG working solution (MGU7418, China) was then added to treat cells for 60 min in a 37 °C incubator. Hoechst (C0030, China) dye was used as a nuclear stain to identify cells in image analysis of 2-NBDG uptake.

### 2.6 Western blotting analysis

As mentioned above, the treated cells were rinsed with ice-cold PBS and then lysed with RIPA buffer (Servicebio, China) containing proteinase and phosphatase inhibitors (Beyotime, China, product no. P1050) prior to centrifugation at 12000 rpm for 10 min at 4 °C. Afterwards, the supernatants were collected for measurement of the protein concentration with BCA Protein Assay Kit (ThermoFisher Scientific). Protein extracted from the treated cells were separated by SDS-PAGE and transferred to a PVDF membrane. Membranes were blotted with primary antibodies recognizing p-mTOR (Zen BioScience, China, product no.381548, 1: 700), mTOR (Proteintech, China, product no.28273-1, 1: 5000), HIF-1α (Proteintech, China, product no. 20960-1, 1: 6000), RUNX2 (Bioss, China, product no.bs-1134R, 1:400), SP7 (Abcam, USA, product no.ab209484, 1:800) and β-actin (Proteintech, China, product no.66009-1, 1: 60000). Then, the membranes were washed with TBST and further incubated with the horseradish peroxidase (HRP)-conjugated anti-rabbit (Zen BioScience, China, product no. 511203, 1:5000) or anti-mouse (Zen BioSciences, China, product no. 511103, 1:5000) secondary antibodies at room temperature for 1 h. Finally, the membranes were incubated with enhanced chemiluminescence (ECL) working solution (Tanon, China) and then visualized on the Tanon 5200Multi imaging system. Densitometry was performed using ImageJ software.

### 2.7 Synthesis of hydrogels with varying compositions

The sodium alginate powder (Sigma-Aldrich, USA, 180947) was mixed with deionized water to prepare a 2 wt% sodium alginate gel (Alg). Subsequently, 2 wt% Ca-Zn cross-linked alginate gels (Ca = 11.8 mM, Zn = 20 μM), 2 wt% Mg-Zn cross-linked alginate gels (Mg = 20.8 mM, Zn = 20 μM), and a 2 wt% Ca-Mg cross-linked alginate gel (Ca = 11.8 mM, Mg = 20.8 mM) were prepared by mixing various concentrations of divalent cations.

### 2.8 Animal surgery

Forty healthy mature male SD rats (age: 3 months; weight: ±250 g) were used. The animal experiments were conducted in accordance with the approved protocols (SYSU-IACUC-2022-001991) from the Animal Care and Use Committee at Sun Yat-sen University. The rats were anesthetized via intraperitoneal injection using 1 % sodium pentobarbital at a dose of 5 ml/kg, and the left femur was carefully depilated and sterilized with iodophor. Subsequently, a 1-cm incision was made on the lateral side of the femur and the femoral shaft was exposed through blunt muscle dissection. A 1.5 mm drill bit was used to create a hole in the bone shaft, which was accompanied by saline irrigation for reduction of tissue heating. Afterwards, the defect was filled with the injected hydrogels containing different divalent cations. Finally, the wound was sutured layer by layer.

### 2.9 *In vitro* release rate of hydrogels

1 mL of the hydrogels containing the divalent cations was injected into a tube with the addition of 9 mL of distilled water. 100 μL of extract, which was taken from each tube at 6 h, 12 h, 1, 3, 5, 7, 10, and 14 days, was used for measurement of Mg, Ca, and Zn ion concentrations by ICP-MS (iCAP Qc, Thermo Fisher Scientific).

### 2.10 *In vivo* degradation rate of hydrogels

To evaluate the *in vivo* stability and degradation rate of the hydrogel, the hydrogel was physically mixed with the fluorescence dye Cy5, and then injected into rats to track their degradation at 0, 1, 2, 3, 4, 5, 6, 7, and 10 days post-surgery by using an *in vivo* imaging system (IVIS) (PerkinElmer IVIS Lumina Series III).

### 2.11 Micro-computed tomography analysis

The harvested femora were scanned and imaged using a SkyScan 1276 Micro-CT (Bruker, Kontich, Belgium). The parameters, with an isotropic voxel size of 10 μm and a spatial resolution 100 μm, voltage of 85 kV, and current of 200 μA, were set for sample scanning. The data were analyzed using CTAn version 1.9 software (Bruker), and a three-dimensional model was generated in CTVox version 2.0 (Bruker). New bone, reconstructed from bone defects, was quantified for data analysis using bone volume fraction (BV/TV), trabecular bone number (Tb. N), trabecular bone thickness (Tb. Th), bone mineral density (BMD), and trabecular separation (Tb. Sp).

### 2.12 Decalcified bone for histological analysis

Samples were collected at 3 and 6 weeks post-surgery, and the soft tissue attached to the bone was then carefully removed prior to fixation in a solution of 4 % paraformaldehyde. After micro-CT scanning, the samples were subsequently immersed in a decalcification solution containing 10 % EDTA. The decalcification solution was refreshed every two days until complete demineralization was achieved. Then, the specimens were dehydrated using a 30 % sucrose solution and embedded in OCT for cryo-sectioning. Finally, 10 μm thick coronal sections of the decalcified femora were obtained using a freezing microtome and prepared for histological staining. For hematoxylin-eosin (H&E) staining, the sectioned tissues were stained with hematoxylin for a duration of 3 min and subsequently counterstained with eosin for 1 min. The resulting images were acquired using light microscopy (BDS400, CQOPTEC).

For immunofluorescence staining, the sectioned tissue was subjected to antigen retrieval at room temperature, followed by permeabilization with 0.5 % Triton X-100 and blocking with 5 % BSA. Afterwards, the sections were incubated by primary antibodies at 4 °C overnight and secondary antibodies at room temperature for 1 h. Finally, nuclei were stained using 4′, 6-diamidino-2-phenylindole (DAPI), which was followed by images captured with a laser confocal microscope (Nikon AX). The following primary and secondary antibodies were utilized: ChAT (PA5-29653, Thermo Fisher Scientific), TH (AB152, Sigma-Aldrich), CGRP (14959S, Cell Signaling Technology), CD31 (bs-0195R, Bioss), Endomucin (sc-65495, santacruz) and anti-rabbit IgG (H + L) labeled with Alexa Fluor 647.

### 2.13 Statistical analysis

Data are presented as means ± standard deviations (mean ± SD). The independent student's t-test was applied for statistical analysis of data in two groups while one-way ANOVA combined with Tukey's post-hoc correction was used for multiple comparisons of data in more than two groups. In terms of histologic scores, the Kruscal-Wallis test was applied to compare the values in different groups. The sample size was calculated using G∗Power software [38]. The values of type I error α and power (1-β) were set as 0.05 and 0.8, respectively. All statistical analysis were performed with GraphPad Prism software version 8.0 (GraphPad Software, Inc., CA, USA).

## 3 Results

### 3.1 Higher tolerance limits of Mg^2+^, Ca^2+^, or Zn^2+^ in the dual cation groups than the single cation

**Fig. 1:**
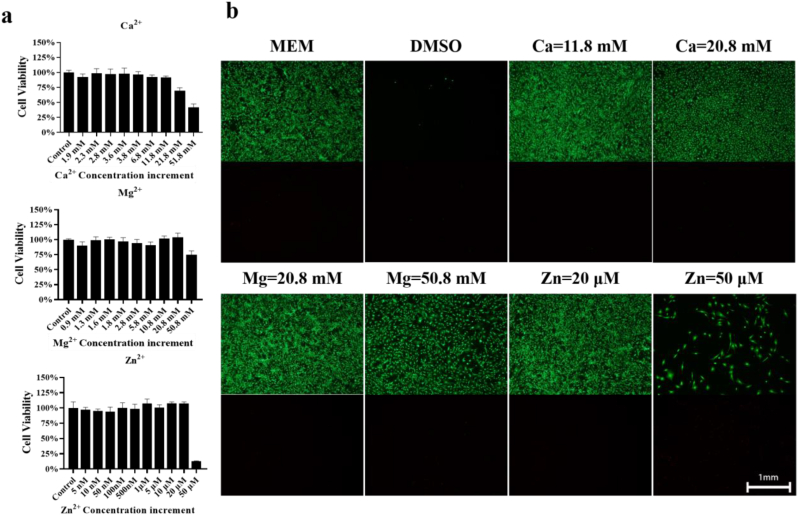
Effects of Ca^2+^, Mg^2+^, and Zn^2+^ with different levels on cytotoxicity of BMSCs. (a) CCK8 assay of BMSCs incubated by different concentrations of Ca^2+^, Mg^2+^, or Zn^2+^ in the complete medium. n = 6. (b) Representative fluorescence images showing Calcein-AM (live cells in green)/PI (dead cells in red) staining of BMSCs incubated by different concentrations of Ca^2+^, Mg^2+^, or Zn^2+^ in the complete medium. The cell culture medium with the addition of DMSO was set as the positive control. n = 3. Scale bar: 1 mm.

In this study, we first determined the cytotoxicity of various levels of individual and combined dual ions. Prior to cytotoxicity testing, we measured if the addition of the divalent cations altered pH values of the cell culture medium. Although the stock solutions of the divalent cations showed weak acidic due to the hydrolysis reactions, the cell culture medium with the divalent cations at their final concentrations showed neutral (Fig. S1). As shown in Fig. 1a and Fig. S2, there was no negative effects on cell viability for a single Ca^2+^ not more than 11.8 mM. The maximum tolerated level of a single Ca^2+^ without causing cell death was 11.8 mM. Similarly, the maximum tolerated levels of a single Mg^2+^ and Zn^2+^ without causing cell death were 20.8 mM and 20 μM, respectively. The fluorescence staining for Live/Dead cells revealed that a remarkable decrease of live cells was observed after treatment by Ca^2+^ higher than 11.8 mM, Mg^2+^ over 20.8 mM, or Zn^2+^ higher than 20 μM (Fig. 1b).

**Fig. 2:**
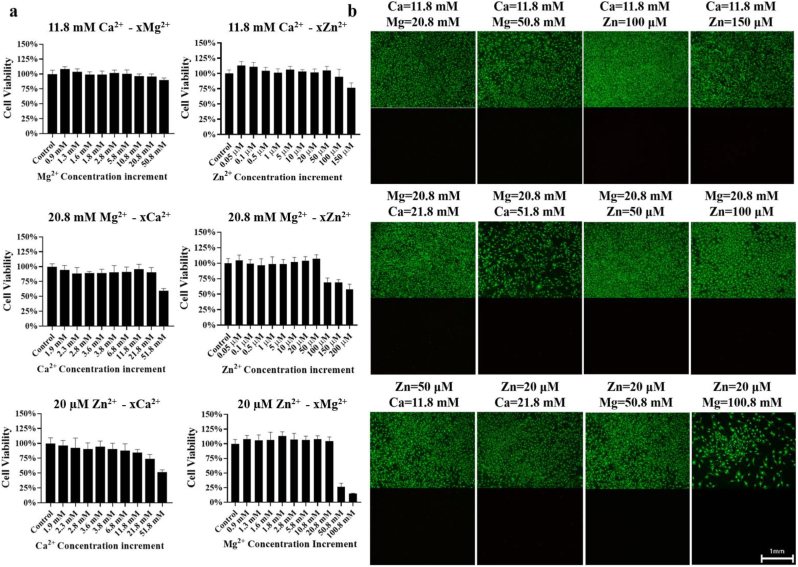
Effect of combination of dual ions composed of Ca-x Mg, Ca-x Zn, Mg-x Ca, Mg-x Zn, Zn-x Ca, and Zn-x Mg on cytotoxicity of BMSCs. x indicates a series of concentrations. (a) Cell viability of BMSCs after culture with combined dual ions with a series of concentrations through CCK-8 assay. n = 6. (b) Representative fluorescence images showing Calcein-AM (live cells in green)/PI (dead cells in red) staining of BMSCs incubated by a series of dual ion systems. n = 3. Scale bar: 1 mm.

As the presence of other ions may affect the tolerance limits of a single ion by cells, the effects of combined dual ions, including Ca-x Mg, Ca-x Zn, Mg-x Ca, Mg-x Zn, Zn-x Ca, and Zn-x Mg binary ion systems, on cytotoxicity of BMSCs were also studied. As shown in Fig. 2a and Fig. S2, the presence of higher Ca^2+^ (11.8 mM) dramatically increased the maximum tolerated dose of Mg^2+^ from 20.8 mM to 50.8 mM and Zn^2+^ from 20 μM to 50 μM, which did not negatively affect cell viability. Similarly, the addition of higher Mg^2+^ (20 mM) remarkably increased the maximum tolerated dose of Ca^2+^ from 11.8 mM to 21.8 mM and Zn^2+^ from 20 μM to 50 μM. Interestingly, the addition of higher Zn^2+^ (20 μM) did not induce alteration of the maximum tolerated doses of Ca^2+^ and Mg^2+^. The fluorescence staining for Live/Dead cells revealed that a remarkable decrease of live cells was observed after treatment by Ca^2+^ higher than 11.8 mM, Mg^2+^ over 20.8 mM, or Zn^2+^ higher than 20 μM (Fig. 2b). Of note, the extended culture (7 days) of BMSCs in the medium with the addition of the divalent cations at their maximum tolerated levels did not induce cytotoxicity (Fig. S3).

### 3.2 The divalent metal cations enhance adhesion and migration of BSMCs through promoting expression levels of Integrin-β1 and TGF-β1

**Fig. 3:**
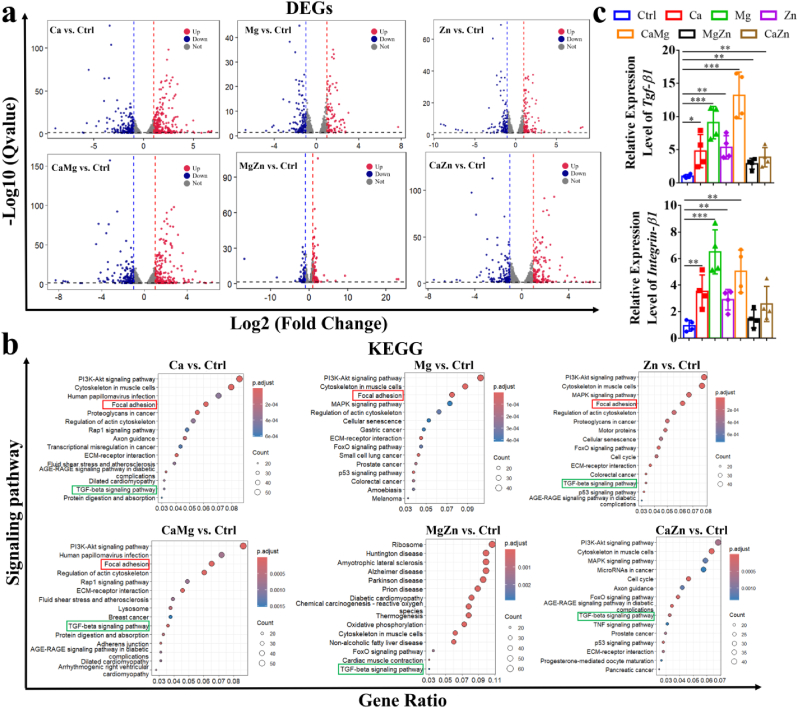
RNA-seq analysis of BMSCs treated with or without the divalent metal cations and measurement of the related gene expression levels. (a) Volcano plot of gene expression in the divalent cation group versus the control group. (b) KEGG pathway enrichment in the divalent cation group versus the control group. (c) Quantitative analysis of the related gene expression levels of BMSCs treated with or without the divalent cations. ∗P < 0.05, ∗∗P < 0.01, ∗∗∗P < 0.001. n = 3 biological replicates.

The RNA-sequencing (RNA-seq) work was then performed in BMSCs to investigate if the direct treatment of the divalent cations influence cell molecular events, like adhesion and migration. According to the results of differential expression genes (DEGs) in BMSCs (Fig. 3a), the top 15 involved items were summarized in the Kyoto Encyclopedia of Genes and Genomes (KEGG) analysis (Fig. 3b). Of note, the Focal adhesion pathway and the TGF-beta signaling pathway were involved. Different from other divalent cations, the divalent cations composed of Mg and Ca ions affected molecular events of BMSCs through modulation of both the Focal adhesion pathway and the TGF-beta signaling pathway, which was further verified by quantitative real-time PCR results in Fig. 3c, indicating the favorable effects of Mg and Ca ions on adhesion and migration of BMSCs (Fig. S4).

### 3.3 The divalent metal cations improve osteogenesis of BSMCs via promotion of aerobic glycolysis of osteoblasts after differentiation initiation by CGRP

**Fig. 4:**
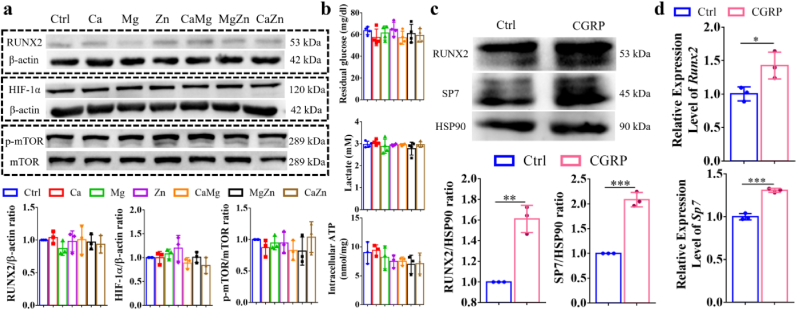
Effects of the divalent metal cations and CGRP on osteogenesis and aerobic glycolysis of undifferentiated BMSCs. (a) Measurement of glucose and lactate concentrations in the medium as well as intracellular ATP production in the undifferentiated BMSCs. (b) Quantitative analysis of osteogenic differentiation and aerobic glycolysis related proteins of the BMSCs treated by the divalent metal cations in the cell culture medium without OIM addition. (c) Relative expression levels of osteogenic differentiation related proteins (c) and genes (d) of BMSCs with or without CGRP treatment in the absence of OIM. ∗P < 0.05, ∗∗∗P < 0.001. n = 3 biological replicates.

As shown in Fig. 4a, the treatment of BMSCs, which exhibited tri-lineage differentiation potential (Fig. S5), by the divalent cations or their combination in the cell culture medium without OIM addition did not affect RUNX2 expression level. The glucose metabolic switch can be regulated by several pathways including HIF-1α and p-mTOR/mTOR. For instance, HIF-1α can induce an increased expression of glycolytic enzymes while the activation of mTOR favors aerobic glycolysis [29], so the expression level of HIF-1α and p-mTOR/mTOR ratio in the lysed cells were quantified. In the absence of OIM, the divalent cation treatment did not significantly affect the expression levels of HIF-1α and p-mTOR/mTOR ratio. Consistently, the divalent cation treatment did not significantly influence the concentrations of glucose, lactate, and intracellular ATP without OIM addition (Fig. 4b). As the divalent cations significantly increased production of CGRP from the sensory neurons (Fig. S6), we then investigated if the CGRP could initiate osteogenic differentiation of BMSCs. Interestingly, the CGRP treatment significantly upregulated gene and protein expression levels of RUNX2 and SP7 of BMSCs without OIM addition, indicating that CGRP could initiate the osteogenesis of BMSCs (Fig. 4c and d).

**Fig. 5:**
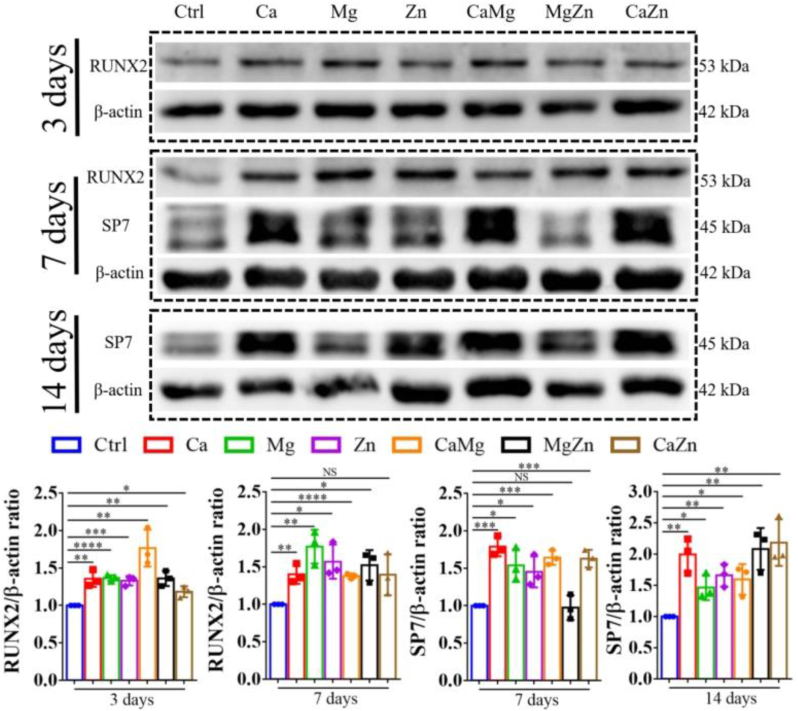
Effects of the divalent metal cations on osteogenesis and expression levels of aerobic glycolysis related proteins of BMSCs at different differentiation stages after the addition of OIM. ∗P < 0.05, ∗∗P < 0.01, ∗∗∗P < 0.001, ∗∗∗∗P < 0.0001. n = 3 biological replicates.

**Fig. 6:**
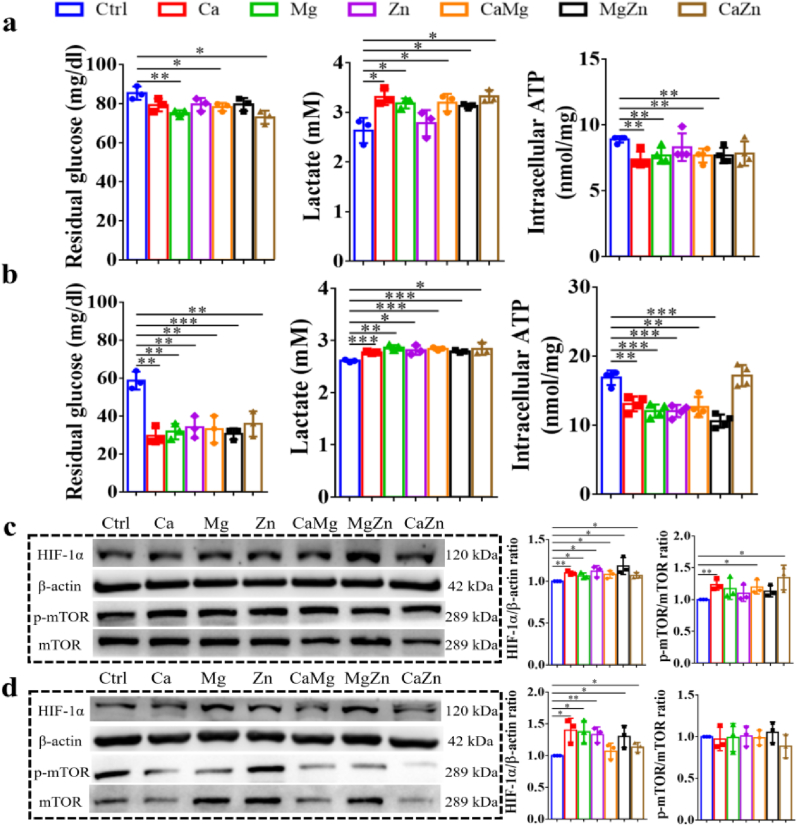
Effects of divalent metal cations on aerobic glycolysis of differentiated BMSCs at different differentiation stages. Measurement of glucose and lactate concentrations in the medium as well as intracellular ATP production in the differentiated BMSCs at 7 days (a) and 14 days (b). Quantitative analysis of aerobic glycolysis related proteins of the differentiated BMSCs at 7 days (c) and 14 days (d). ∗P < 0.05, ∗∗P < 0.01,∗∗∗P < 0.001. n = 3 biological replicates.

**Fig. 7:**
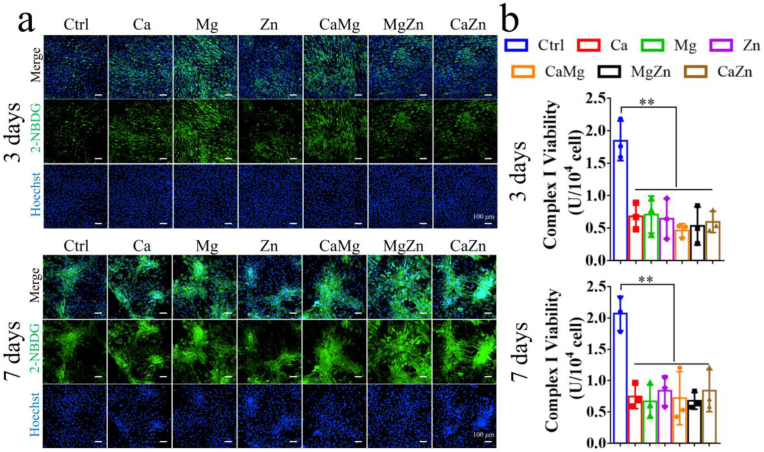
The effects of the divalent cations on glucose uptake and mitochondrial respiration in the differentiated BMSCs. (a) Representative images showing fluorescence intensity of 2-NBDG for measurement of glucose uptake in the BMSCs at 3 and 7 days after osteogenic differentiation. Scale bars: 100 μm. (b) Measurement of mitochondrial Complex I activity of BMSCs at 3 and 7 days after osteogenic differentiation. ∗P < 0.01. n = 3 biological replicates.

However, after the addition of OIM in the cell culture medium, the divalent cations significantly promoted expression levels of RUNX2 and SP7 at different differentiation stages (Fig. 5), implying that CGRP laid the foundation for the divalent cations exerting pro-osteogenic effects on BMSCs. Of note, the treatment of the divalent cations significantly promoted aerobic glycolysis of the differentiated BMSCs. As shown in Fig. 6a and b, compared to the normal medium, the medium with the addition of single or dual cations significantly reduced glucose concentration in the medium while increased lactate production. Simultaneously, the intracellular ATP levels were also significantly decreased in the cation-treated BMSCs after osteogenic differentiation relative to the untreated BMSCs. As expected, the expression level of HIF-1α and the p-mTOR/mTOR ratio significantly increased in the cation-treated BMSCs compared to the untreated BMSCs (Fig. 6c and d). In addition, as shown in Fig. 7a, the addition of the divalent cations dramatically enhanced the fluorescence intensity of 2-NBDG in BSMCs after osteogenic differentiation at 3 and 7 days, indicating improved glucose uptake in the divalent cation-treated BMSCs. Consistently, the addition of the divalent cations significantly reduced mitochondrial Complex I activity (Fig. 7b), implying compromised mitochondrial respiration in the differentiated BMSCs after treatment by the divalent cations.

### 3.4 The divalent metal cations promote bone regeneration and type H vessel formation in the bone defect model

**Fig. 8:**
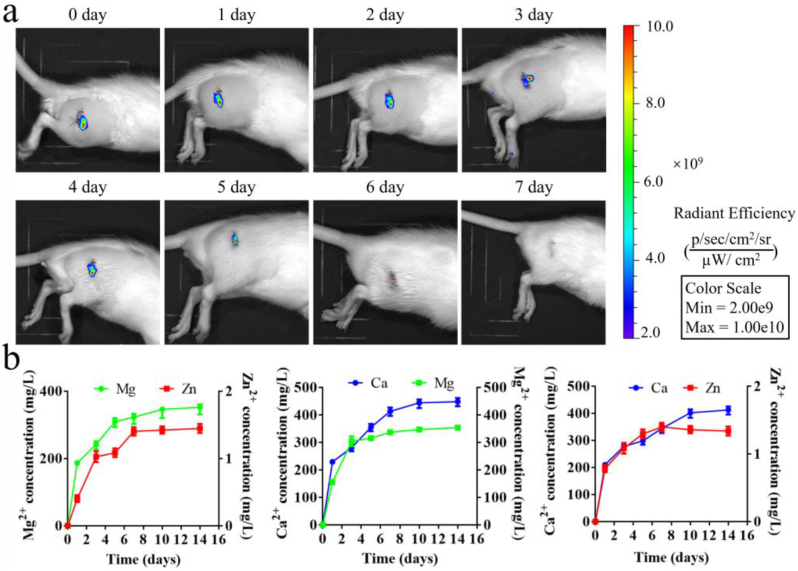
Release patterns of the divalent cations over time. (a) Representative images showing *in vivo* degradation behavior of the hydrogels in bone defects of rats under IVIS. (b) Cumulative release amount of the divalent cations from the hydrogels over time. n = 3 biological replicates.

**Fig. 9:**
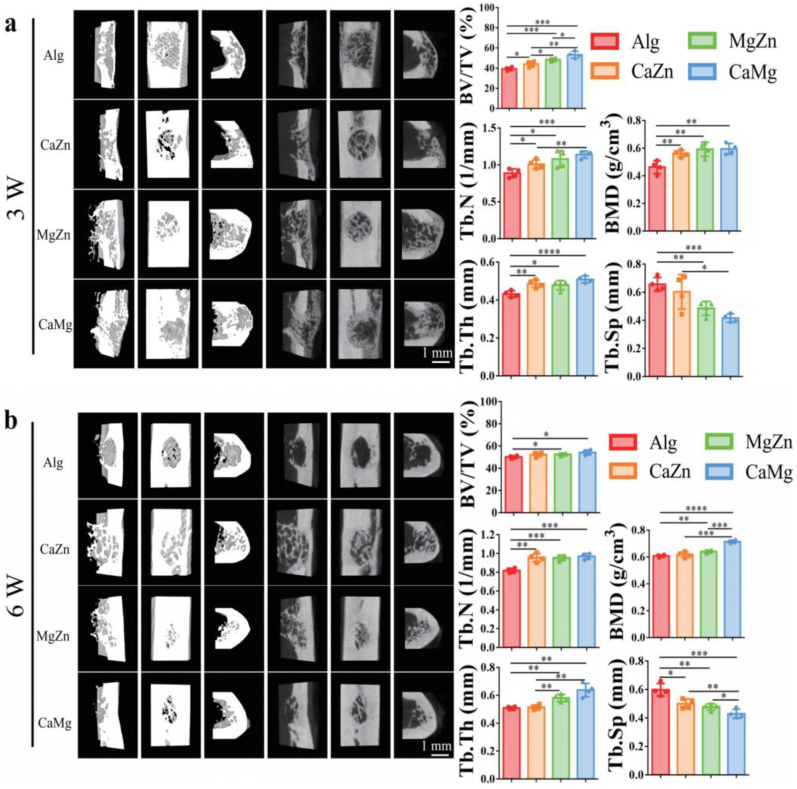
Micro-CT analysis of the newly formed bone tissue in the femoral defects in the rats. Representative 3D reconstructed bone defect models in different view directions and the corresponding 2D tomography images as well as the quantitative analysis of bone structure parameters including BV/TV, Tb. N, BMD, Tb. Th, and Tb. Sp in the regenerated bone in the rats after treatment with alginate (Alg) or the dual metal cations at 3 weeks (a) and 6 weeks (b) post-surgery. ∗P < 0.05, ∗∗P < 0.01, ∗∗∗P < 0.001, ∗∗∗∗P < 0.0001, n = 4 biological replicates. Scale bar: 1 mm.

In order to magnify the biological functions of the divalent cations, the alginate hydrogels containing different dual cations, i.e. Mg-Zn, Ca-Zn, or Ca-Mg, were injected into the drilled holes in the femora in rats. As reported by Yeung's work [39], the effect of Mg^2+^ on bone remodeling was time-dependent. For instance, the Mg^2+^ favored bone regeneration at the exposure time of 7 days while impaired bone regeneration at the exposure time of 14 days, which may be ascribed to TRPM7 kinase-mediated immunomodulation in macrophage. Therefore, both of the *in vitro* release rate of the divalent cations and the *in vivo* degradation rate of the hydrogels were measured. As shown in Fig. 8a and b, the hydrogels, labeled by the fluorescence dye Cy5, was completely degraded within one week while the *in vitro* cumulative amount of the divalent cations, i.e. Mg^2+^, Zn^2+^, and Ca^2+^ ions, nearly reached a plateau after 7 days, which indicates the release pattern of the divalent cations in the hydrogels may well support bone regeneration. Then, the effects of the released cations on the bone regeneration were assessed by micro-CT analysis. As shown in Fig. 9a and b, the results of micro-CT analysis showed that the dual metal cations significantly promoted new bone formation in the rats relative to the control group in terms of bone structure parameters like bone volume fraction (BV/TV), trabecular number (Tb. N), bone mineral density (BMD), trabecular thickness (Tb. Th), and trabecular separation (Tb. Sp) at both 3 weeks and 6 weeks post-surgery. Of note, the combination of Ca and Mg ions exhibited the best performance in promotion of bone regeneration.

**Fig. 10:**
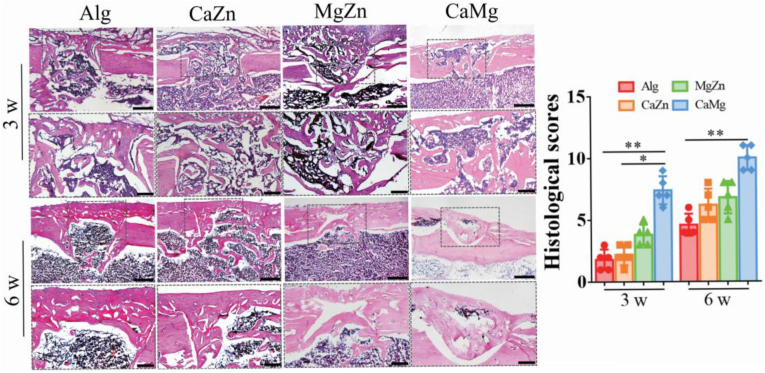
Representative histological images and quantitative analysis of histological scores showing the bone defect healing in the rats with treatment by the blank Alg or the dual metal cations at 3 weeks and 6 weeks post-surgery. ∗P < 0.05, ∗∗P < 0.01, n = 5 biological replicates. Scale bar: 500 μm.

**Fig. 11:**
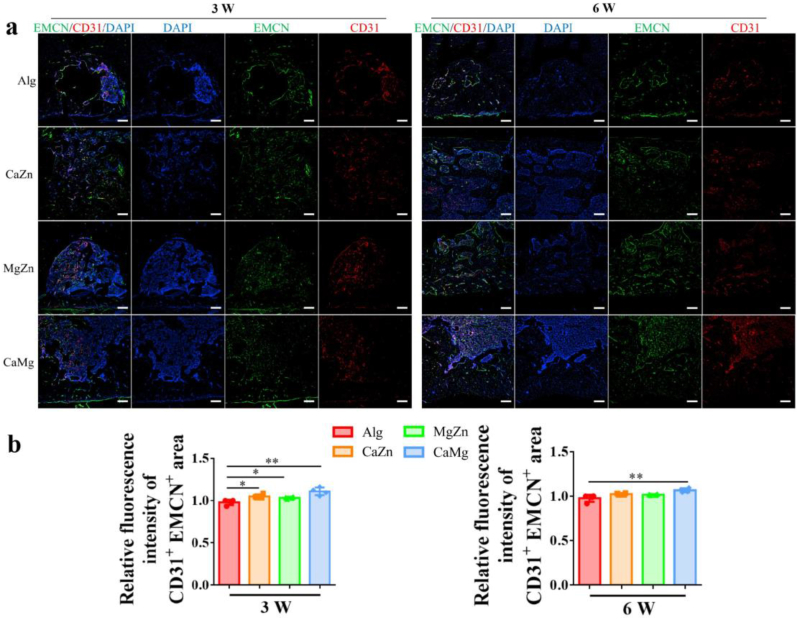
Representative images (a) and quantitative analysis (b) of type H vessels (CD31^hi^EMCN^hi^) in the bone defects in the rats with treatment of the blank Alginate (Alg) or the dual metal cations at 3 weeks and 6 weeks post-surgery. ∗P < 0.05, ∗∗P < 0.01, n = 4 biological replicates. Scale bar: 200 μm.

Consistently, the histological assessment revealed that the dual cations significantly improved the healing quality in terms of histological scores, including bone formation, cortical integrity, and bone remodeling, in the bone defects in the rats compared to the alginate treated rats at 3 weeks and 6 weeks post-surgery (Fig. 10). As reported, there are rich type H vessels (CD31^hi^EMCN^hi^) distributed in the periosteum [40], contributing to bone regeneration due to the existence of a large number of osteoprogenitor cells around type H vessels. As shown in Fig. 11a and b, the treatment of dual metal cations significantly increased type H vessel formation in the bone defects relative to the alginate treatment at 3 weeks post-surgery. Interestingly, although the CaZn or MgZn treatment did not significantly increase type H vessel formation relative to the alginate treatment at 6 weeks post-surgery, the CaMg treatment still significantly increased type H vessel formation in the bone defects.

### 3.5 The divalent metal cations upregulate activities of sensory and parasympathetic nervous systems while tune down activity of sympathetic nervous system

**Fig. 12:**
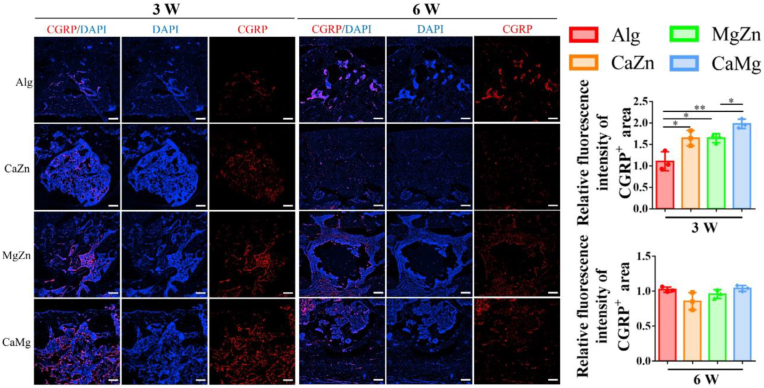
Representative immunofluorescence images showing CGRP positive area in the bone defects and the quantitative analysis of the relative fluorescence intensity of CGRP positive area in the rats with treatment by the blank Alg or the dual metal cations at 3 weeks and 6 weeks post-surgery. ∗P < 0.05, ∗∗P < 0.01, n = 3 biological replicates. Scale bar: 200 μm.

The neurotransmitters, like CGRP released from sensory nerves [1]and Ach produced by parasympathetic nervous system [41], favor osteogenic differentiation of MSCs and thereby promote bone regeneration. As shown in Fig. 12, the dual metal cation treatments significantly increased fluorescence intensities of CGRP positive area in the bone defects relative to the alginate treatment in rats at 3 weeks post-surgery. Of note, compared to the MgZn and the CaZn groups, the CaMg group exhibited best performance in enhancement of CGRP expression levels.

**Fig. 13:**
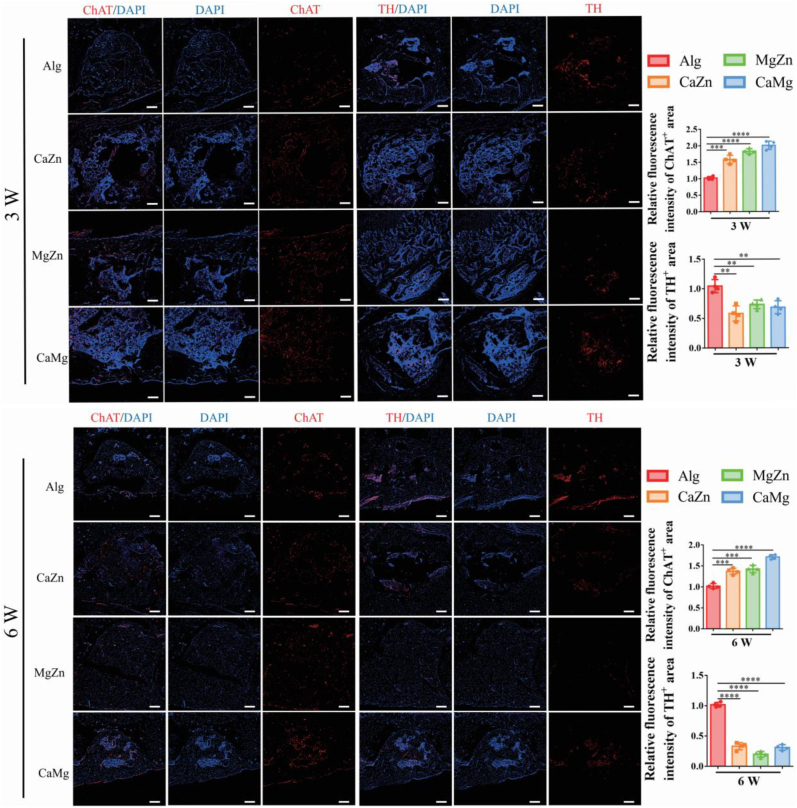
Representative immunofluorescence images showing ChAT and TH positive area in the bone defects and the quantitative analysis of the relative fluorescence intensities of ChAT and TH positive area in the rats with treatment by the blank Alg or the dual metal cations at 3 weeks and 6 weeks post-surgery. ∗∗∗P < 0.001, ∗∗∗∗P < 0.0001, n = 4 biological replicates. Scale bar: 200 μm.

Tyrosine hydroxylase (TH) is a rate-limiting step enzyme that plays a vital role in the biosynthesis of catecholamines like dopamine and NE [42], so the expression level of TH positively correlates with NE production. Choline acetyltransferase (ChAT) is a transferase enzyme responsible for the synthesis of Ach, a PSN derived neurotransmitter [43]. As shown in Fig. 13, the dual metal cations significantly upregulated the relative fluorescence intensities of ChAT positive area while significantly downregulated the relative fluorescence intensities of TH positive area in the bone defects relative to the blank alginate at both 3 weeks and 6 weeks post-surgery, indicating promotion of PNS activity while inhibition of SNS activity after treatment of dual metal cations in the rats.

## 4 Discussion

The divalent metal cations, like Mg, Zn, and Ca, as essential nutrients in human body, exert favorable effects on new bone formation, so the cations containing hydrogels [44], scaffolds [45], inorganic coatings [46], and the biodegradable metal fixators or implants [12]have been extensively developed in orthopaedics in recent years. Of note, the modulation pathway of the divalent metal cations in osteogenesis of MSCs still remains largely unknown. How to unveil the underlying mechanism behind the pro-osteogenic differentiation of MSCs induced by the divalent cations from indirect or direct pathways is a key step for the development of high efficient biological therapeutic agents or implants in clinics. In terms of the indirect pathway, we discovered that the divalent cations, Mg^2+^, Ca^2+^, Zn^2+^, and their dual metal cations, upregulated activities of sensory nerves and PNS while downregulated activity of SNS, thereby contributing to increased bone and type H regeneration in the bone defects. In terms of the direct pathway, the divalent cations alone could not initiate osteogenic differentiation of MSCs and affect their aerobic glycolysis. However, the OIM or neurotransmitters (e.g. CGRP) could initiate osteogenic differentiation of MSCs without influencing their aerobic glycolysis. Of note, after initiation of osteogenic differentiation, the MSCs with the treatment of the divalent cations exhibited significantly higher osteogenesis and improved aerobic glycolysis, indicating that the indirect pathway laid down the foundation for the direct pathway in the modulation of osteogenic differentiation of MSCs.

As the sodium alginate hydrogels exhibit many advantages including satisfactory biocompatibility, biodegradability, and easy gelation, thereby acting as a potential drug delivery system in tissue engineering [47,48]. In terms of bone regeneration, sodium alginate has been widely used to load growth factors [49], antibiotics [50], calcium incorporated nanoparticles [51], and cells [52] to favor bone formation. However, native sodium alginate is a bioinert material [53], so the presence of sodium alginate itself rather than the divalent cations would not affect bone regeneration. Nevertheless, the stiffness of a hydrogel can affect the differentiation of stem cells into different lineages. For instance, the hydrogels with stiffness between 2.5 and 5 kPa favor adipogenesis while the hydrogels with stiffness between 11 and 30 kPa facilitates osteogenesis [54]. As the shear moduli of the hydrogels in this work were less than 100 Pa, the effects of the hydrogels on osteogenic differentiation of MSCs can be excluded (Fig. S7). In our previous work, we verified that Mg ions favored the transportation of CGRP containing synaptic vesicles toward nerve terminals of neuron via ATP-facilitated actin polymerization and higher production of CGRP in dorsal root ganglion (DRG) [1]. In addition to the direct stimuli in sensory neuron for secretion of CGRP, the divalent metal cations increased PGE2 production from CD68^+^ macrophages [14], thereby leading to activation of EP4 signaling in the sensory nerve endings and ultimately inducing the release of CGRP [55]. Consistently, compared to the blank alginate treatment, the divalent cations significantly increased CGRP levels in the defect zone in rats (Fig. 12). As CGRP is beneficial for the osteogenic differentiation of osteoblasts, the SLIT3, which is produced from osteoblasts and favors type H vessel (CD31^hi^EMCN^hi^) formation, also contributed to significant increase of type H vessels in the defect zone in the cation-treated groups relative to the control group (Fig. 11). Type H vessels are densely surrounded by Runx2^+^ and Osterix^+^ osteoprogenitors [56], so the increased type H vessel formation favored bone regeneration. Therefore, the crosstalk between nerve-osteogenesis-angiogenesis induced by the divalent metal cations indicates the complexity of modulation pathways for the pro-osteogenic effects in bone formation.

However, it is still unclear which pathway predominantly upregulates CGRP secretion and how the divalent cations affect CGRP biosynthesis in DRG neurons. Our data confirmed that the production of the pro-osteogenic factor CGRP in the sensory neurons, isolated from DRG of rats, was significantly upregulated after the treatment of the divalent cations (Fig. S6), indicating the direct effect of the divalent cations on neurotransmitter release. In addition to the direct effect, the divalent cations also exerted indirect effect on CGRP secretion. For instance, PGE2 could depolarize sensory axons and then trigger action potentials by activating the sodium channel Nav1.8 and the calcium-activated chloride channel Anoctamin 1 (ANO1) [57], thereby eliciting neurotransmitter release for initiation of osteogenic differentiation of MSCs. Consistently, the deletion of EP4 in the sensory nerve completely abolished the cation-induced bone formation [14].

Simultaneously, the activation of PGE2/EP4 in DRG neurons causes phosphorylation of cAMP response element binding protein (CREB) in the ventromedial hypothalamus (VMH), tuning down sympathetic tones [14], so the expression level of TH was downregulated in the cation-treated groups compared to the control group (Fig. 13). As the activation of the PGE2/EP4 signaling axis was positively correlated with Ach production [58], the expression level of ChAT was significantly upregulated in the cation-treated groups relative to the control group (Fig. 13). In the indirect pathway, the joint contributions of CGRP (upregulation), Ach (upregulation), and NE (downregulation) in the osteogenic differentiation of MSCs largely determine bone regeneration in the femoral defects. Encouragingly, it was consistent with the findings of histological scores and Micro-CT analysis, which was selected as the primary outcome for the calculation of the sample size (Fig. S8).

In addition to the indirect pathway, different divalent metal cations exert diverse effects on undifferentiated and differentiated MSCs via a direct manner. In RNA-seq analysis, the divalent cations activated the Focal adhesion signaling and the TGF-beta signaling pathway in BMSCs (Fig. 3). Of note, the activation of TGF-beta 1-dependent signaling pathway favors migration of bone marrow mesenchymal stem cells (BMSCs) to the injury site for tissue repair [59], while the activation of Focal adhesion-dependent signaling pathway, e.g. Integrin-FAK pathway, facilitates cell adhesion at the target region for tissue regeneration [60]. Importantly, our previous study confirmed that the Mg^2+^ ions increased TGF-beta 1 levels and FAK activity, which was accompanied with enhanced bone formation [61]. It indicated the direct biological function of the divalent cations may also affect bone regeneration.

**Fig. 14:**
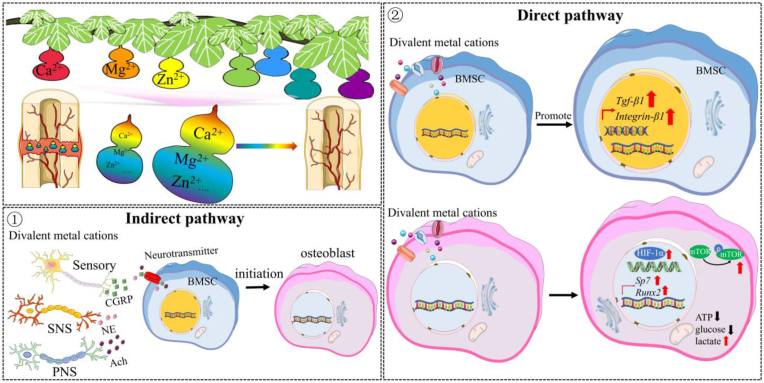
Schematic diagram depicting the underlying mechanism behind the repair contributions of the divalent metal cations in bone regeneration. The divalent metal cations promote the production of the pro-osteogenic neurotransmitters, such as CGRP from sensory nerves and Ach from PNS, while inhibit the biosynthesis of NE from SNS, a neurotransmitter negatively affects osteogenesis of MSCs, contributing to improved bone formation in an indirect pathway. In terms of the direct pathway, the divalent cations upregulate the gene expression levels of *Tgf-β1* and *Integrin-β1* of BMSCs. In addition, as these neurotransmitters initiate osteogenic differentiation of MSCs, the divalent metal cations exert positive effects on aerobic glycolysis of differentiated MSCs for improved osteogenic differentiation potential. Importantly, the combination of different divalent metal cations integrate their diverse biological functions, fusing together into a superpower like “Diamond Brother” in a Chinese animation to promote bone regeneration more efficiently.

Nevertheless, once the osteogenic differentiation of BMSCs was initiated under OIM stimuli, these divalent cations exerted pro-osteogenic effects on the differentiated BMSCs (Fig. 5), which was accompanied by the enhanced aerobic glycolysis (Fig. 6, Fig. 7). It was reported that activation of canonical Wnt/β-catenin signaling upregulated pyruvate dehydrogenase kinase 1 (Pdk1), a key metabolic enzyme involved in aerobic glycolysis through regulating the flux of pyruvate from the cytoplasm into the mitochondria, leading to enhanced aerobic glycolysis [27]. Encouragingly, the divalent cations favored the activation of canonical Wnt/β-catenin signaling [[62], [63], [64]], thereby contributing to promoted aerobic glycolysis, which subsequently improved osteogenesis of BMSCs (Fig. 14). Interestingly, although the divalent cations alone could not initiate osteogenesis of BSMCs and affect glucose metabolism without OIM addition (Fig. 4), CGRP could initiate osteogenesis of BMSCs (Fig. 4 and Fig. S9), which enabled the divalent cations to exert pro-osteogenic effects on the differentiated BMSCs through upregulation of aerobic glycolysis.

However, there were still some limitations for this study. As the rate of bone turnover differs between humans and rats [65], a large animal model with similar bone remodeling process is necessary to extrapolate our findings from the preclinical model to clinics. Nevertheless, as a result of the concern on femoral shaft fracture, the defect size in rats may not be large enough to mimic large bone defects in patients, so a model with a critical-sized segmental bone defect is required to verify our findings prior to clinical trials.

## 5 Conclusion

The divalent metal cations promoted bone regeneration via the direct and the indirect pathways. First, the divalent cations may favor BMSC migration and adhesion through upregulation of gene expression levels of *Tgf-β1* and *Integrin-β1*. Second, the neurotransmitters rather than the divalent cations could initiate osteogenesis of BMSCs, which facilitates the metabolism switch from oxidative phosphorylation towards aerobic glycolysis in the presence of the divalent cations, thereby contributing to improved osteogenic differentiation capability of the differentiated BMSCs. Last, the divalent metal cations promote the production of the pro-osteogenic neurotransmitters, such as CGRP from sensory nerves and Ach from PNS, while inhibit the biosynthesis of NE from SNS, a neurotransmitter negatively affects osteogenesis of BMSCs, ultimately leading to improved bone formation in an indirect pathway. Collectively, the divalent cations promote bone regeneration via regulation of sensory-autonomic nervous systems and enhancement of aerobic glycolysis-driven osteogenesis of differentiated MSCs initiated by neurotransmitters.

## CRediT authorship contribution statement

**Ying Luo:** Software, Methodology, Investigation, Formal analysis. **Baoyi Liu:** Writing – original draft, Methodology, Formal analysis. **Yashi Qiu:** Methodology, Investigation, Formal analysis. **Lichen Li:** Methodology, Formal analysis. **Fan Yang:** Investigation, Formal analysis. **Chao Zhang:** Supervision, Funding acquisition, Conceptualization. **Jiali Wang:** Writing – original draft, Funding acquisition, Conceptualization.

## Ethics approval and consent to participate

The animal experiments were conducted in accordance with the approved protocols (SYSU-IACUC-2022-001991) from the Animal Care and Use Committee at Sun Yat-sen University.

## Declaration of Competing Interests

We wish to confirm that there are no known conflicts of interest associated with this publication and there has been no significant financial support for this work that could have influenced its outcome.
