# Supplementary material for: Divalent metal ions enhance bone regeneration through modulation of nervous systems and metabolic pathways
Source: Bioact Mater. 2025 Feb 12;47:432–47. doi: 10.1016/j.bioactmat.2025.01.034 (PMC11872643; doi:10.1016/j.bioactmat.2025.01.034)
Supplement: Multimedia component 1 [file mmc1.docx]

**Divalent metal cations favor bone regeneration through promoting aerobic glycolysis of differentiated MSCs and modulating activity of sensory and autonomic nervous systems**

Materials and Methods

**Three Lineage Differentiation of BMSCs**

The BMSCs in passage 3 underwent three lineage differentiation experiments in vitro, namely, osteogenesis, adipogenesis and chondrogenesis. The cells were seeded in six-well plates at a density of 2 × 10^5^ cells per well, cultured in growth medium until close to 80% confluent, and then appropriate induction reagents were added.

Osteogenic differentiation: The cells were stimulated for 3 weeks in standard culture medium supplemented with 50 μM ascorbic acid, 10 mM sodium β-glycerophosphate, and 100 nM dexamethasone, and the presence of calcium deposition was revealed using an Alizarin Red staining kit (Solarbio, G1450).

Chondrogenic differentiation: Cells were stimulated for 3 weeks in standard culture medium supplemented with 50 μg/mL ascorbic acid, 10 ng/mL human recombinant transforming growth factor β3 (Novoprotein, CJ44), 0.1 mg/mL sodium pyruvate, 1% insulin-transferrin-selenium, and 100 nM dexamethasone, and proteoglycan deposition was visualized by Alcian blue staining.

Adipogenic differentiation: Standard culture medium was supplemented with 1 μM dexamethasone, 0.5 mM isobutylmethylxanthine (IBMX), 200 μM indomethacin and 10 ug/mL human recombinant insulin to induce culture for 3 weeks, and lipid droplets were visualized by Oil Red O staining.

**Primary culture of DRG neurons and metal ion stimulation of CGRP release**

In brief, we harvested DRG from rats and isolated neurons through removal of the adherent nerve fibers and then digestion in a mixture of collagenase A (1.25 mg/mL) and dispase II (2.5 mg/mL) by gently pipetting in an incubator at 37°C for 30 min. After that, 3 volumes of cell culture medium were added to terminate digestion prior to filtration through a 70 µm filter for removal of tissue debris. Then, the cell pellets, which were collected after centrifuge at 900 rpm for 5 min, were pipetted with the high glucose medium and seeded in a 6-well plate coated with rat tail collagen. After 8 hours for cell adhesion, the culture medium containing cytosine β-D-arabinofuranoside (Ara-C), nerve growth factor (NGF) 2.5S, and glial cell-line derived neurotrophic factor (GDNF), which was refreshed every two days, was added for the replacement of the original medium. The supernatant of the cell culture medium, with or without additional supplementation of the divalent cations, was collected after 24 h treatment of neurons for measurement of CGRP by ELISA.

**Rheological property**

The hydrogel samples were placed on a rotating plate with a diameter of 25 mm and a gap of 1 mm, and the rheological properties were tested using a rheometer (MCR 302e, Anton Paar GmbH, Austria). The temperature of the rotating plate was controlled at 37 °C, and shear strains from 0.01% to 100% were performed. Secondly, a frequency sweep test from 0.1 to 100 rad/s was performed at a fixed shear strain (1%). Finally, a time sweep was performed at a controlled frequency of 0.16 Hz (normal cell sensing frequency).

**Table S1**. The pros and cons of CCK-8, MTT, LDH and Live/Dead assays.

| Assay | CCK-8 | MTT | LDH | Live/Dead |
| --- | --- | --- | --- | --- |
| Principles | Measurement of dehydrogenase activity in live cells | Measurement of mitochondrial activity in live cells | Measurement of LDH released from damaged cell membranes | Measurement of intracellular esterase activity and plasma membrane |
| Sensitivity | High | Low | High | High |
| Stability | High | Medium | Medium | High |
| Cytotoxicity | Low | High | Low | Low |
| Easy to use | Easy | Medium | Easy | Easy |
| Phenomenon | Yellow | Purple | Yellow | Live cells green and dead cells red |


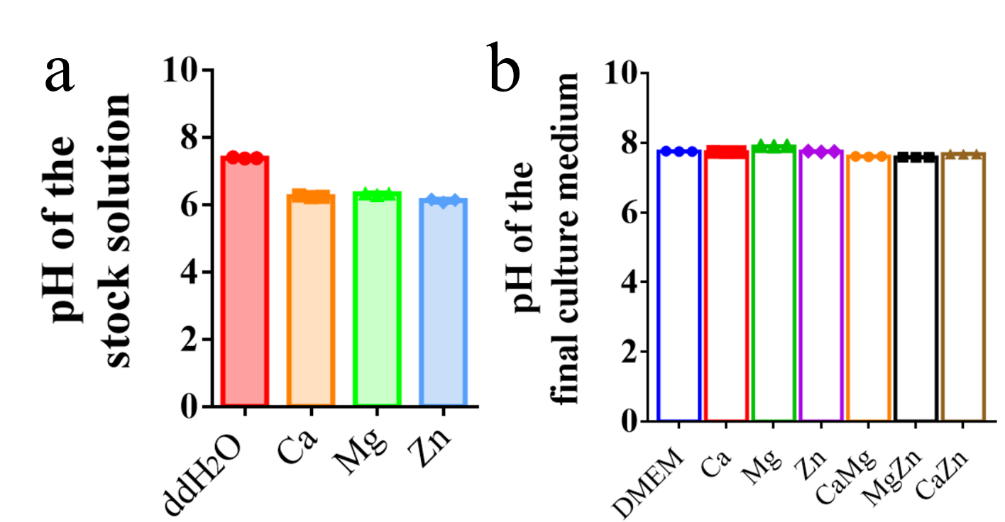


**Figure S1** The effects of divalent cations on pH values in the stock solutions (a) and the cell culture medium at their final concentrations (b).


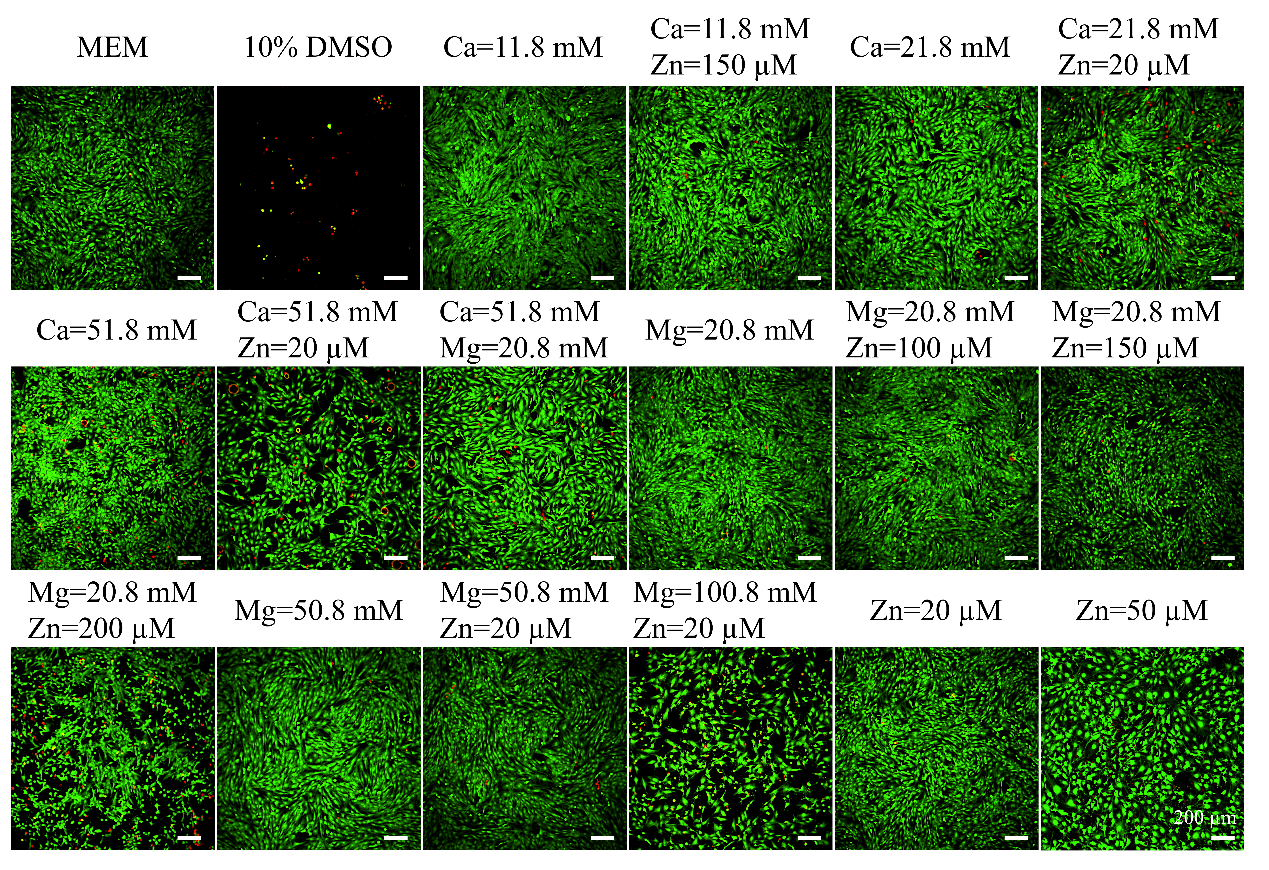


**Figure S2** Cytotoxicity test of BMSCs cultured in the medium with or without the addition of the divalent cations by using Live/Dead assay after 24 hours.


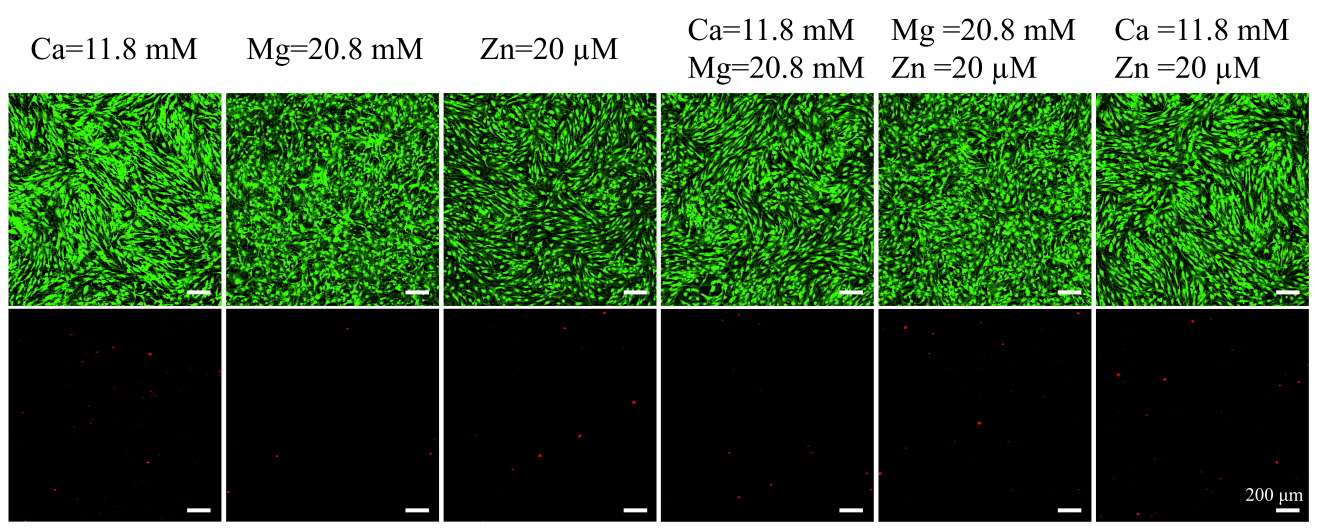


**Figure S3**. Cell viability in divalent cation culture for 7 days.


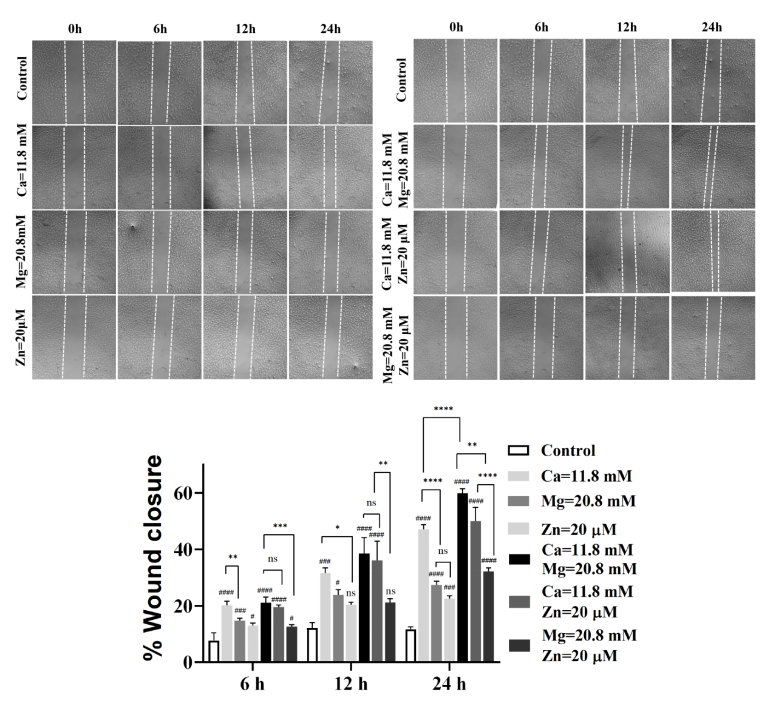


**Figure S4.** Measurement of cell migration rate of BMSCs treated by a single ion or combined dual ion systems. ns: not significant, *: p<0.05, **: p<0.01, ***: p<0.001, ****: p<0.0001. #: p<0.05 vs control, ###: p<0.001 vs. control, ####: p<0.0001 vs. control. n=3.


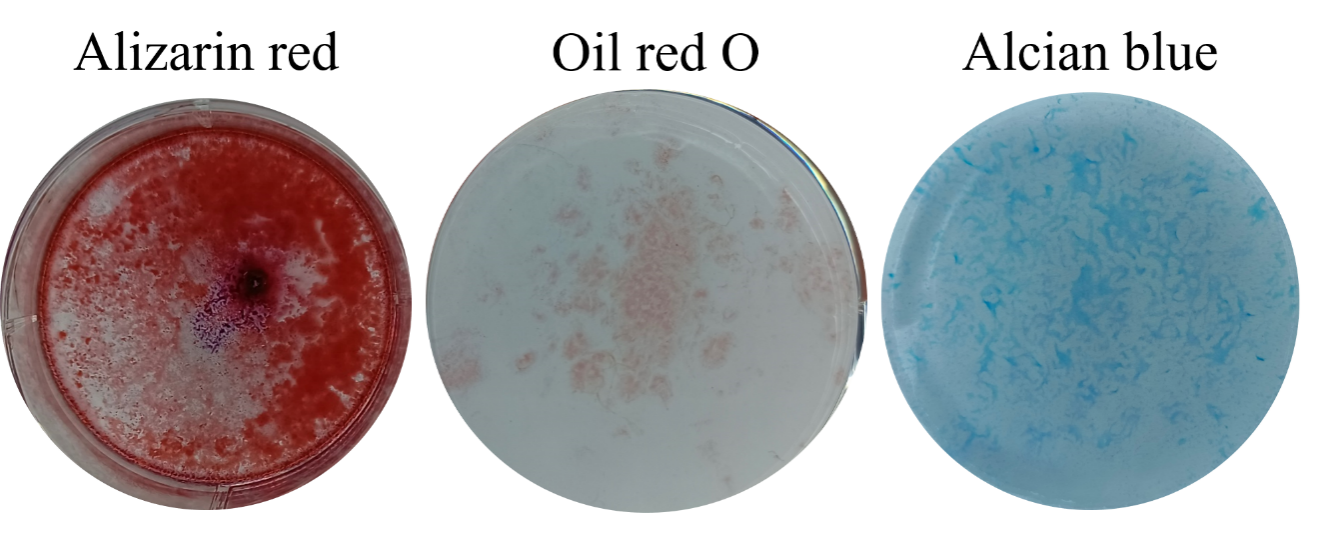


**Figure S5**. Representative images showing tri-lineage differentiation potential of the isolated BMSCs by Alizarin red, Oil red O and Alcian blue staining.


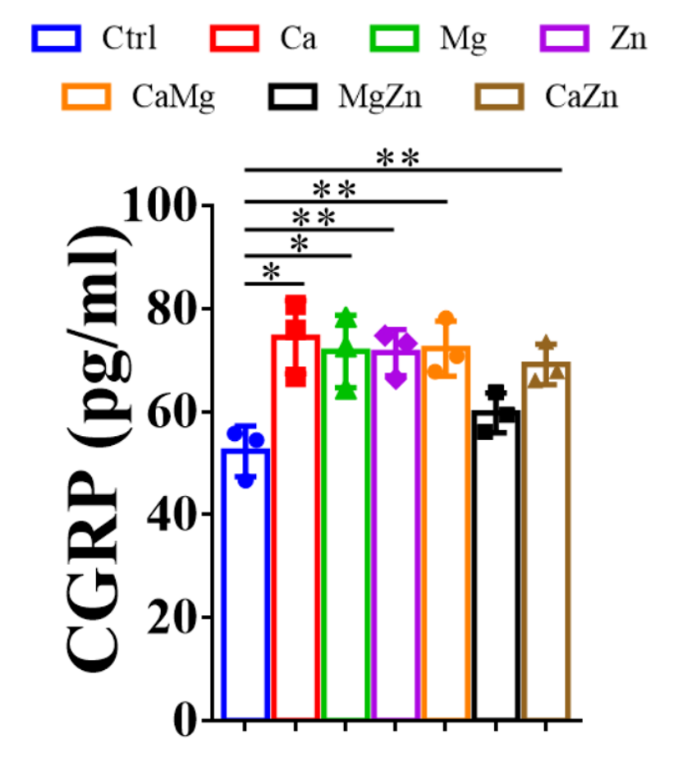


**Figure S6**. The effects of the divalent cations on CGRP production in sensory neurons by ELISA. *P<0.05, **P<0.01, n=3 biological replicates.


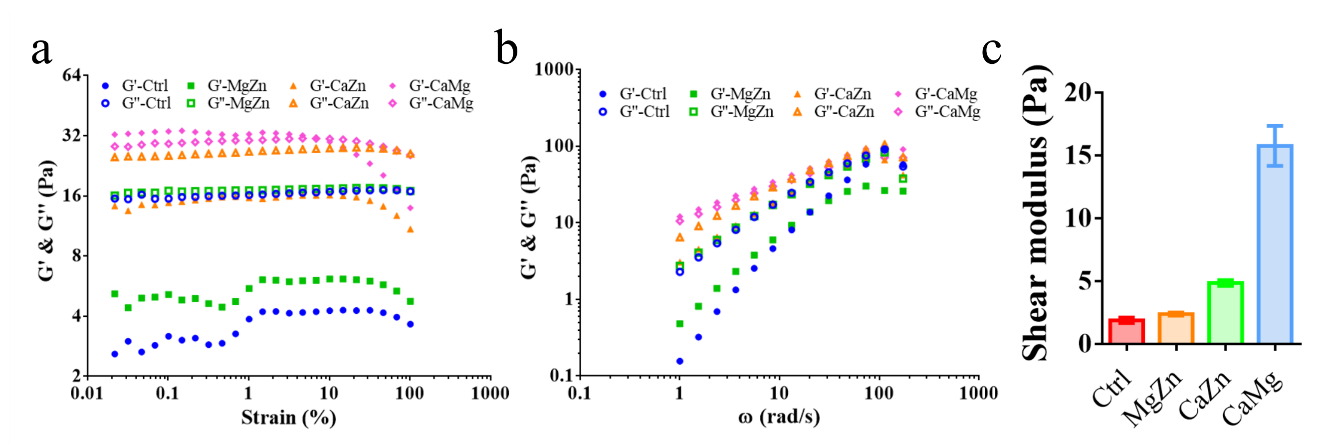


**Figure S7**. The rheological properties of the hydrogels. (a) Strain sweep of the hydrogels. (b) Frequency sweep of the hydrogels. (c) The shear modulus of the hydrogels with or without the addition of the divalent cations. n=3 biological replicates.


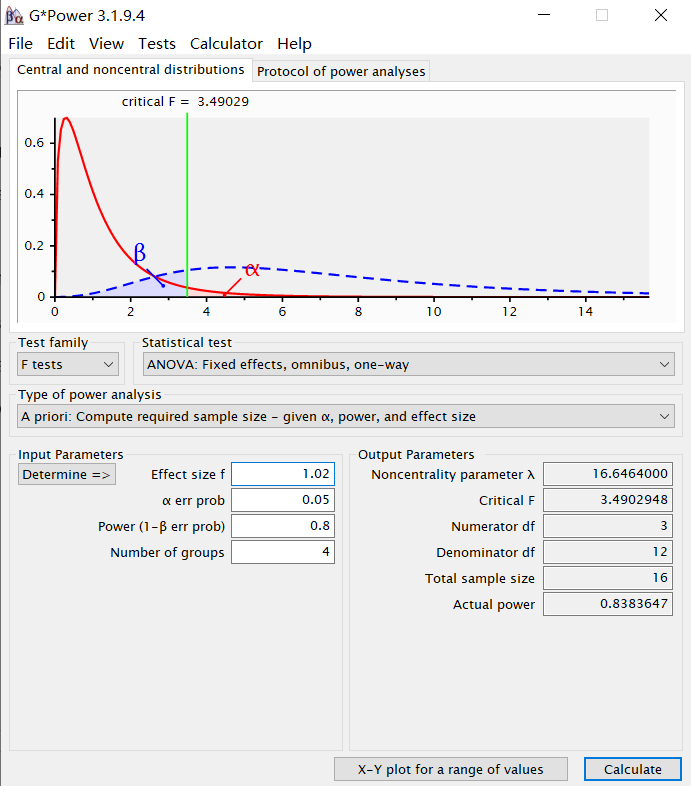

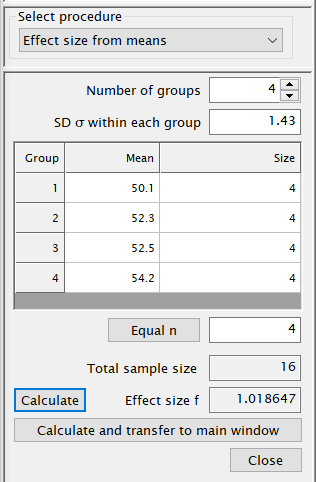


**Figure S8**. Sample size calculation procedure.


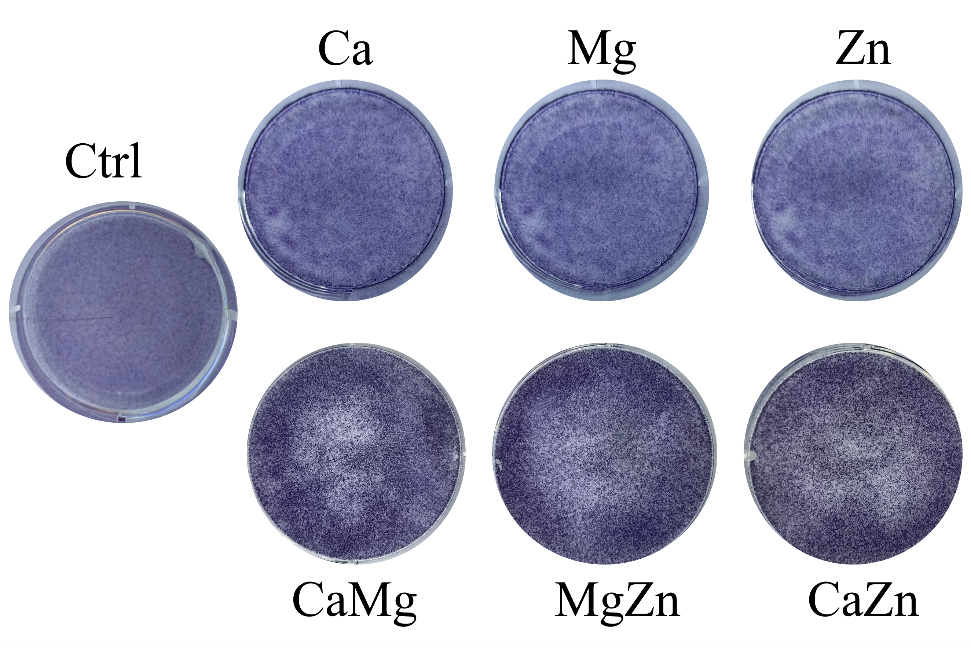


**Figure** **S9** Representative images showing the effects of the divalent cations on osteogenic differentiation of BMSCs by ALP staining.
